# Characteristics of Fundal Changes in Fundus Tessellation in Young Adults

**DOI:** 10.3389/fmed.2021.616249

**Published:** 2021-04-26

**Authors:** Hanyi Lyu, Qiuying Chen, Guangyi Hu, Ya Shi, Luyao Ye, Yao Yin, Ying Fan, Haidong Zou, Jiangnan He, Jianfeng Zhu, Xun Xu

**Affiliations:** ^1^Shanghai Eye Disease Prevention and Treatment Center, Shanghai Eye Hospital, Shanghai, China; ^2^Shanghai General Hospital, Shanghai Jiaotong University School of Medicine, Shanghai, China; ^3^Shanghai Jiaotong University School of Medicine, Shanghai, China; ^4^Shanghai Engineering Center for Visual Science and Photo medicine, Shanghai Jiaotong University School of Medicine, Shanghai, China; ^5^Ophthalmology Department of Peking University People's Hospital, Beijing, China

**Keywords:** fundus tessellation, myopia, fundal alteration, choroidal thickness, scleral thickness

## Abstract

**Purpose:** To explore the characteristics and associated factors of fundus tessellation, especially the alternation of choroidal thickness among different degrees of tessellated fundus in young adults.

**Design:** Cross-sectional, population-based study.

**Methods:** A total of 796 students were included in the study and underwent comprehensive ophthalmic examinations, including anterior segment examinations and swept-source optical coherence tomography (OCT) measurements. The degree of tessellated fundus was assessed by fundus photographs applying an early treatment of diabetic retinopathy study grid to evaluate the location of fundus tessellation and then divided into five groups. The topographic variation and factors, tilted disc ratio, parapapillary atrophy (PPA), retinal thickness (ReT), choroidal thickness (ChT), and subfoveal scleral thickness (SST) related to tessellated fundus were analyzed.

**Results:** Compared to normal fundus, tessellated fundus had a lower spherical equivalent (SE) (*p* < 0.0001), worse best-corrected visual acuity (BCVA)(*p* = 0.043), longer axial length (AL) (*p* < 0.0001), thinner retina (*p* < 0.0001), thinner (*p* < 0.0001) choroid, and thinner sclera in center fovea (*p* = 0.0035). Among all subfields of macular and peripapillary regions, center fovea and macula-papillary region showed the most significant decrease in choroidal thickness. The proportion of fundus tessellation significantly increased with lower body weight index (BMI) (*p* = 0.0067), longer AL (*p* < 0.0001), larger PPA(*p* = 0.0058), thinner choroid (*p* < 0.0001), and thinner sclera (*p* < 0.0001).

**Conclusions:** Eyes showed more severe myopic morphological alternation with the increasement of proportion of fundus tessellation to the center fovea, including a significant decrease in both choroid and scleral thickness. Choroidal thinning may progress most rapidly in the macula-papillary region as fundus tessellation approaches to the center fovea.

## Introduction

The high prevalence of myopia and high myopia in young adults worldwide, especially in East and Southeast Asia, has led to a significant public health burden of visual impairment and blindness (1–8). Recently, a revised classification system for myopic maculopathy has been proposed to standardize the definition among epidemiological studies (6, 9). According to this International Photographic Classification and Grading System for myopic maculopathy (META-PM) classification, as well as previous studies, tessellated fundus has been defined as the visualization of large choroidal vessels at the posterior fundus pole, which is the first stage of pathological myopia (PM) retinopathy (category 1) (6, 9, 10). Some tessellated fundus can progress to diffuse atrophy and macular atrophy, which cause severe and irreversible impairment of visual acuity, while others could be stable at this stage for long-term (9, 11). The underlying reasons for this discrepancy remain unknown.

Fundus photographs are common in clinical practice, and can be easily accessed for clinical observation. Factors associated with fundus tessellation in elderly population have been studied using fundus photographs (12, 13), however, age-based choroidal thinning and age-related retinopathy can be interference factors for examining fundus tessellation in pathological myopia (14–17).

To investigate the characteristics associated with fundus tessellation during early stage of pathological myopia, we recruited 828 university students of all grades in Shanghai University, of which 796 students were included as participants in this study. In this cross-sectional study, we measured optical parameters, including parameters of anterior segment, axial length (AL), peripapillary atrophy (PPA), retinal thickness (ReT), choroidal thickness (ChT) and subfoveal scleral thickness (SST), and analyzed their relationships with tessellated fundus diagnosis and severity.

## Methods

### Setting and Participants

This cross-sectional population-based study was approved by the ethics committee of Shanghai General Hospital, Shanghai Jiao Tong University, Shanghai, China, and followed the tenets of the Declaration of Helsinki. All participants understood the study protocol and provided signed informed consents. The study was registered at www.clinicaltrials.gov (No.NCT03446300).

The subjects were randomly selected from the students attending the Shanghai University in October 2016 (18). Age and gender were recorded and height, weight, heart rate, systolic (SBP) and diastolic (DBP) blood pressures were measured for each participant. A detailed medical history was recorded for each participant. All participants underwent comprehensive ophthalmic examinations, including refractive error assessment using an autorefractor machine (model KR-8900; Topcon, Tokyo, Japan), measurement of best-corrected visual acuity (BCVA) and intraocular pressure (IOP, Full Auto Tonometer TX-F; Topcon, Japan), slit-lamp biomicroscopy, and color fundus photograph. Central corneal thickness, lens thickness, anterior chamber depth (ACD) and AL were measured using optical low-coherence reflectometry (Aladdin; Topcon, Japan). Subjective refraction was performed by two trained optometrists for all participants. BMI is defined as the body mass divided by the square of the body height. Spherical equivalent (SE) was acquired in non-cycloplegic manner and was calculated as the sphere plus half a cylinder. BCVA was converted into the logarithm of minimal angle resolution (logMAR).

The inclusion criteria were as follows: IOP ≤ 21 mmHg; normal anterior chamber angles; normal optic nerve head (ONH) without glaucomatous changes, such as narrowing of neuroretina, increased cup-disc ratio and peripapillary hemorrhage. Participants with a history of ocular or systemic diseases including congenital cataract and glaucoma, hypertension and diabetes; previous intraocular or refractive surgery; and other evidence of retinopathy were excluded. Images acquired with signal strength index ≤60 were excluded for statistical analysis. In general, except for the fundus tessellation associated with myopia, the participants had no other ocular abnormalities. The analysis of correlation between two eyes of 30 randomly selected participants has showed that for parameters including SE, AL, PPA, SST, ChT, GCT, ReT, and the grading of fundus tessellation, correlation is significant at the 0.01 level. Only the right eye of each participant was selected for statistical analyses, for a significant correlation at 0.01 level of major parameters concerned in the study between the two eyes was detected in 30 randomly selected participants using spearman's analysis, including SE, AL, PPA, ReT, ChT, and SST.

### Assessment of Fundus Tessellation, Tilted Disc (TD), and Parapapillary Atrophy Area (PPA)

The diagnostic and degree of fundus tessellation was assessed on the 45° fundus photographs centered on the macula. Retinal photographs centered on the macular and optic disc were acquired from the same SS-OCT, which was fitted with a digital, non-mydriatic retinal camera. The optic disc tilt and the PPA area were calculated from these photographs using Image J version 1.60 software (National Institutes of Health, MD, USA; http://rsb.info.nih.gov/ij/index.html) by two independent, blinded, well-trained observers (Z.H and Z.Z). Average data were used for the final analysis.

The macular area and the peripapillary area were separately analyzed using the macula photographs and the optic disc-centered photographs.

**Figure 1:**
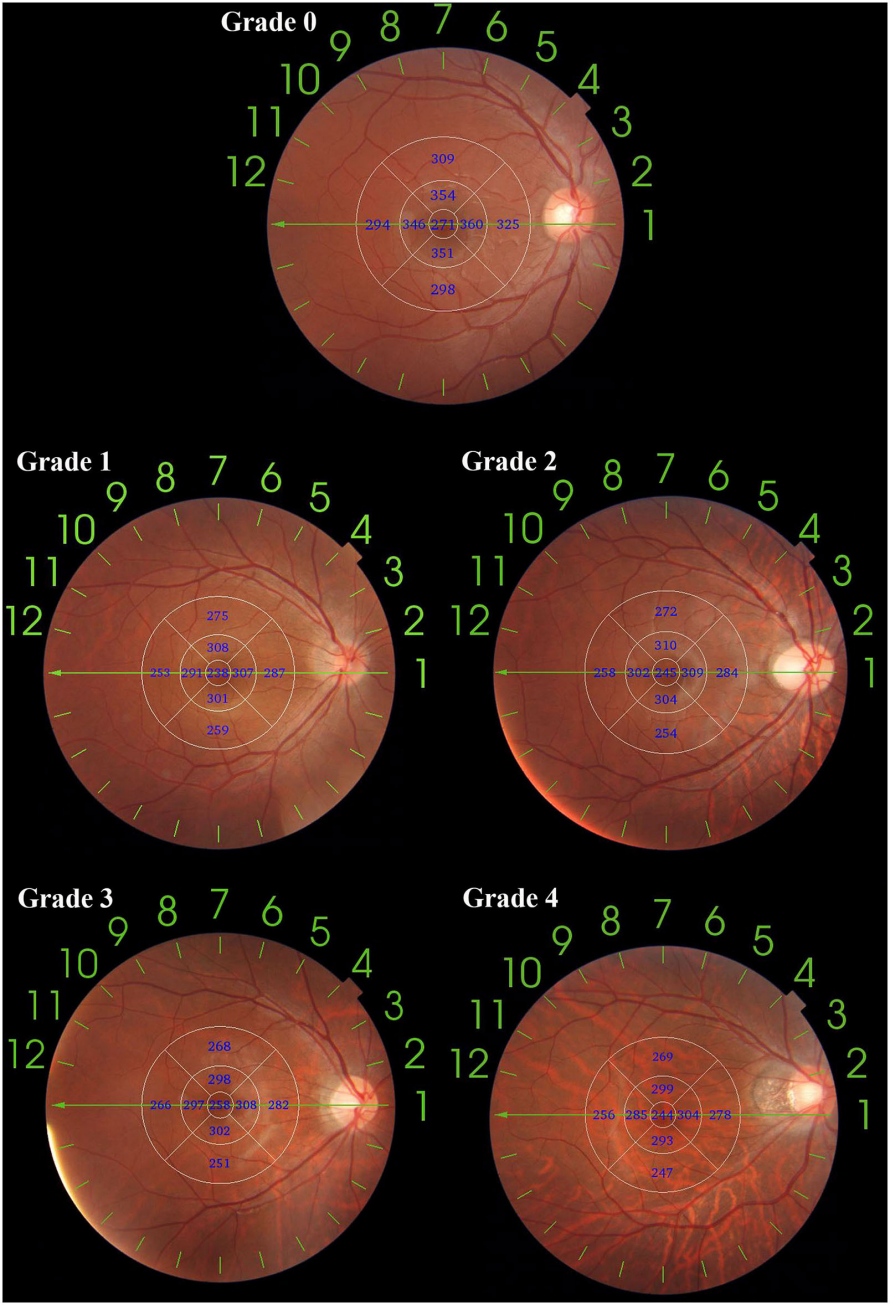
An application of ETDRS grid on fundus tessellation grading. The diameter of center, inner and outer circle are 1, 3, and 6 mm. Eyes with no fundus tessellation were graded as Grade 0; eyes with fundus tessellation without involving the outer circle were graded as Grade 1; eyes with fundus tessellation involving the outer circle of were graded as Grade 2; eyes with fundus tessellation involving the inner circle were graded as Grade 3; eyes with fundus tessellation involving the center circle were graded as Grade 4.

Fundus tessellations is defined by the ophthalmoscopic visualization of the large choroidal vessels. The degree of tessellated fundus was assessed by fundus photographs applying an early treatment of diabetic retinopathy study (ETDRS) grid to evaluate the relative location between fundus tessellation and the fovea, where Grade 0 is defined as no large choroidal vessels visible, Grade 1 is defined as fundus tessellations visible in the posterior pole without involving ETDRS grid, Grade 2 is defined as fundus tessellations visible in the outer circle of ETDRS grid without involving the inner circle, Grade 3 is defined as fundus tessellations visible in the inner circle of ETDRS grid without involving the fovea, and Grade 4 is defined as fundus tessellations visible in the center fovea of ETDRS grid (Figure 1). The fundus tessellation was assessed by a trained examiner (L.H.Y), who was regularly supervised by two experienced ophthalmologists (C.Q.Y, F.Y). To assess the reproducibility of the technique, the images of 100 eyes of 100 participants were randomly selected and graded twice by a trained grader (H.G.Y) in a masked manner at an interval of 2 weeks.

The definition of TD ratio has been previously described as the tilt ratio of minimum-to-maximum disc diameter and a tilted optic disc had a tilt ratio 0.80 or less (19). The optic disc margins, defined as the inner border of the peripapillary sclera rings were lined for measurements (20). The gamma zone peripapillary atrophy was defined as the region between optic disc border and end of Bruch's membrane in previous studies (20–22). Briefly, the area of PPA area was determined as the total number of pixels in a circumferential pattern using the Image J software. Magnification by fundus camera was × 1.4, the total magnification was calculated combined with the magnification factor of Image J system. The area of PPA was converted from pixels into square millimeters. The magnification was corrected for each AL using the Littmann's formula (23).

### Swept-Source Optical Coherence Tomography (SS-OCT) Imaging

The tomography thickness map of the entire macular and disc area (6 × 6 mm) was acquired from an average of four overlapped consecutive scans using SS-OCT (model DRI OCT-1 Atlantis; Topcon), which had a lateral resolution of 10 μm and a depth resolution of 8 μm. The machine had a scanning speed of 100,000 A-scans per second with a 1,050-nm-wave length light source. The scan protocol utilized the 12-line radial scan pattern with a resolution of 1,024 × 12 centered on the fovea and optic disc.

The segmentation of each layer was automatically obtained with the built-in software. ChT was defined as the distance between the Bruch membrane and the choroid-sclera interface. Manual segmentation was performed since the automatic segmentation was inaccurate or led to measurement artifacts. The tomography maps were overlapped to an early treatment diabetic retinopathy study grid (6 × 6 mm) that was focused on the macular and optic disc. The global average thickness of the choroid, retina, ganglion cell layer (GCL), and retinal nerve fiber layer (RNFL) was calculated. The circle placement was manually adjusted, if necessary. All measurements were conducted by a single technician who was experienced in taking OCT images. Images with signal strength index ≤60 were excluded for statistical analysis. The SS-OCT was performed twice for the first 30 participants to assess measurement reproducibility.

Scleral thickness was defined as the vertical distance between the choroidal–scleral interface and the outer scleral border^*^. The posterior scleral border was carefully identified by an experienced technician and reconfirmed by another experienced technician before measuring the lamellar structure, continuity and high reflectivity value of the retrobulbar tissue. To ensure the reproducibility of posterior scleral border, 20 OCT images were randomly selected from the database in advance and the two experienced technicians were asked to outline the posterior scleral border and measure the SST. The intraclass correlation efficient was 0.943 (*P* < 0.001), which indicates a satisfying repeatability of the method. After testing the reproducibility, measurements were performed by two skilled observers blinded to the study. The average of these measurements was calculated and included in the analysis. If the absolute difference between the two measurements were >20 μm for the sclera, 10 μm for the choroid or 20 μm for the whole fundus, the measurements were repeated until the absolute difference was within the set limits.

### Statistical Analysis

The data analyses were performed by SAS 9.3 (Statistical Analysis System, version 9.3; the SAS Institute, Cary, NC, USA). Demographic and ocular characteristics were shown as counts or proportions for categorical data, and as mean ± standard deviation for continuous data. The distribution of all variables was examined for normality using the Kolmogorov-Smirnov test. The ANOVA test was performed to detect differences in demographic and ocular parameters as well as each average layer's thickness between the four groups, as appropriate. Participants' characteristics with and without accessible scleral thickness were compared using the chi-square statistic for proportions and a t-test or Mann–Whitney U test for means or medians as appropriate, using person-specific data. AL, PPA, ReT, ChT, SST were also categorically assessed (in quartiles). Logistic regression models were then performed to investigate the relationship between the proportion of fundus tessellation with ocular and systemic parameters. Parameters with *p* < 0.05 in the partial correlation analysis were included in the multivariate models.

## Results

Among the 828 students enrolled in the study, 11 were excluded due to diffuse chorioretinal atrophy, nine due to other retinopathy, 12 because of IOP > 21 mmHg. Subsequently, 796 students were included in the final analysis.

The general characteristics of the 796 participants and comparison among participants with or without fundus tessellation are listed in Table 1. There were no significant differences in age, sex, BMI, blood pressure, intraocular pressure, anterior chamber depth and lens thickness between the two groups. Eyes with fundus tessellation diagnosis had a lower SE (*p* < 0.0001), worse BCVA (*p* = 0.043), longer AL (*p* < 0.0001), thinner retina (*p* < 0.0001), thinner choroid (*p* < 0.0001), and thinner sclera in center fovea (*p* = 0.0035). Thinner ganglion cell layer (GCL) thickness (*p* < 0.0001), and more TD existence (*p* = 0.0001).

**Table 1:** Comparison of characteristics between participants with or without fundus tessellation^*^.

**Variable**	**Without fundus tessellation**	**With fundus tessellation**	**Statistic value**	***p*-value**
N	78	718		
Age	19.86 ± 2.20	19.82 ± 2.66	0.014	0.905^a^
Sex (male/female)	31/47	333/385	1.248	0.264^b^
BMI (kg/m^2^)	21.14 ± 3.80	20.66 ± 2.85	1.836	0.176^a^
SE	−2.94 ± 2.20	−4.32 ± 2.38	23.86	< 0.0001^a^
BCVA, logMAR	0.11 ± 0.35	0.24 ± 0.57	4.101	0.043^a^
AL	24.46 ± 1.10	25.36 ± 1.08	49.174	< 0.0001^a^
PPA	0.08 ± 0.08	0.17 ± 0.11	41.754	< 0.0001^a^
Average ReT, μm	283.54 ± 11.05	276.27 ± 11.93	26.491	< 0.0001^a^
Average ChT, μm	296.02 ± 54.37	207.60 ± 50.97	208.899	< 0.0001^a^
SST, μm	518.04 ± 37.33	465.51 ± 47.37	14.515	0.0035^a^
TD- no. (%)	29(37.66)	416(58.02)	11.70	0.001^b^

*BMI, body weight index; SE, spherical equivalent; BCVA, best-corrected visual acuity; AL, axial length; PPA, parapapillary atrophy; ChT, choroidal thickness; ReT, retinal thickness; SST, subfoveal sclera thickness; TD, tilted disc*.

* *Numbers displayed are mean ± standard deviation*.

a *Statistical significance was tested using ANOVA test*.

b *Statistical significance was tested using Chi-square test*.

In this study, the definition of non-myopia was AL ≤24 mm, low myopia was AL more than 24 mm but ≤26 mm, and high myopia was AL more than 26 mm. Among the 796 right eyes included in the study, 690 eyes had myopia (86.68%), and 207 eyes had high myopia (26.01%). The percentages of different grades of fundus tessellation in non-myopic, low myopic and high myopic subjects are listed in Table 2. The percentage of eyes with tessellated appearance from grade 1 to grade 4 in non-myopic group, low myopic group and high myopic group were 70.75, 92.55, and 94.69%.The percentage of eyes with grade 3 or grade 4 tessellated appearance in non-myopic group, low myopic group and high myopic group were 24.53, 53.62, and 73.42%.

**Table 2:** Percentages of fundus tessellation in non-myopic, low myopic, and high myopic subjects.

**Grading of fundus tessellation**		**Non-myopia *n* (%)**	**Low myopia *n* (%)**	**High myopia *n* (%)**
Normal fundus	Grade 0	31 (29.25)	36 (7.45)	11 (5.31)
Tessellated fundus		75 (70.75)	447 (92.55)	196 (94.69)
	Grade 1	9 (8.49)	46 (25.14)	6 (2.90)
	Grade 2	40 (37.74)	142 (29.40)	38 (18.36)
	Grade 3	17 (16.04)	174 (36.02)	76 (36.71)
	Grade 4	9 (8.49)	85 (17.60)	76 (36.71)
Total		106 (100)	483 (100)	207 (100)

Comparison of systematic and ophthalmologic parameters among each degree of fundus tessellation is displayed in Table 3. As fundus tessellation approaches to the fovea, there is a significant decrease in BMI (*p* = 0.044), SE (*p* < 0.0001), ReT (*p* < 0.0001), ChT (*p* < 0.0001), SST (*p* < 0.0001), and a significant increase in AL (*p* < 0.0001) and PPA (*p* < 0.0001).

**Table 3:** Comparisons of characteristics among eyes with different degree of fundus tessellation^*^.

**Variable**	**Grade 0**	**Grade 1**	**Grade 2**	**Grade 3**	**Grade 4**	***p*-value**
N	78	61	220	267	170	
Age	19.86 ± 2.20	20.08 ± 2.73	19.64 ± 2.64	19.72 ± 2.44	20.12 ± 2.97	0.360^a^
Sex (male/female)	31/47	39/22	93/127	137/130	64/106	0.001^b^
BMI (kg/m^2^)	21.14 ± 3.80	21.43 ± 3.69	20.54 ± 2.82	20.82 ± 2.81	20.28 ± 2.55	0.044^a^
AL, mm	24.46 ± 1.10	24.96 ± 0.91	24.95 ± 1.02	25.52 ± 1.01	25.81 ± 1.10	<0.0001^a^
SE (D)	−2.94 ± 2.20	−3.70 ± 2.14	−3.44 ± 2.16	−4.72 ± 2.29	−5.04 ± 2.47	<0.0001^a^
BCVA [logMAR (Snellen)]	0.01 ± 0.03	0.02 ± 0.04	0.02 ± 0.06	0.02 ± 0.05	0.03 ± 0.07	0.064^a^
PPA	0.08 ± 0.08	0.10 ± 0.10	0.14 ± 0.10	0.17 ± 0.10	0.22 ± 0.12	<0.0001^a^
Average ReT, μm	283.54 ± 11.05	285.16 ± 11.75	278.04 ± 11.22	274.91 ± 11.86	272.90 ± 11.12	<0.0001^a^
Average ChT, μm	296.02 ± 54.37	275.97 ± 57.35	234.02 ± 39.10	197.2– ± 35.06	165.21 ± 36.96	<0.0001^a^
SST, μm	518.04 ± 37.33	515.38 ± 34.21	478.39 ± 35.17	471.29 ± 39.46	441.70 ± 54.52	<0.0001^a^

*BMI, body weight index; AL, axial length; SE, spherical equivalent; BCVA, best–corrected visual acuity; PPA, parapapillary atrophy; ChT, choroidal thickness; ReT, retinal thickness; SST, subfoveal sclera thickness*.

* *Numbers displayed are mean ± standard deviation*.

a *Statistical significance was tested using ANOVA test*.

b *Statistical significance was tested using Chi–square test*.

**Figure 2:**
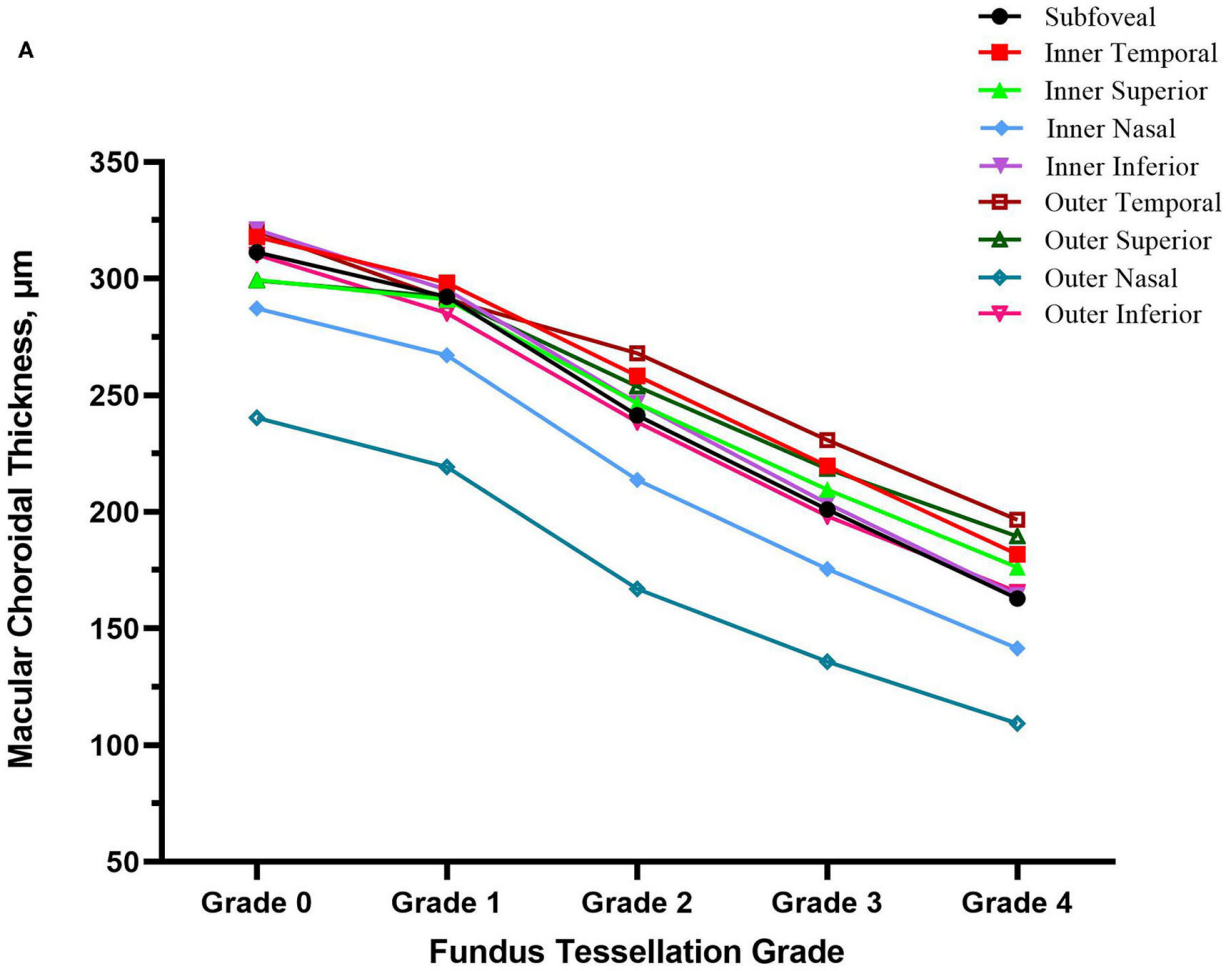
The decrease trend of choroidal thickness in nine subregion of ETDRS grid (subfoveal, inner nasal, inner superior, inner temporal, inner inferior, outer nasal, outer superior, outer temporal, outer inferior) as fundus tessellation approaches to center fovea.

**Figure 3:**
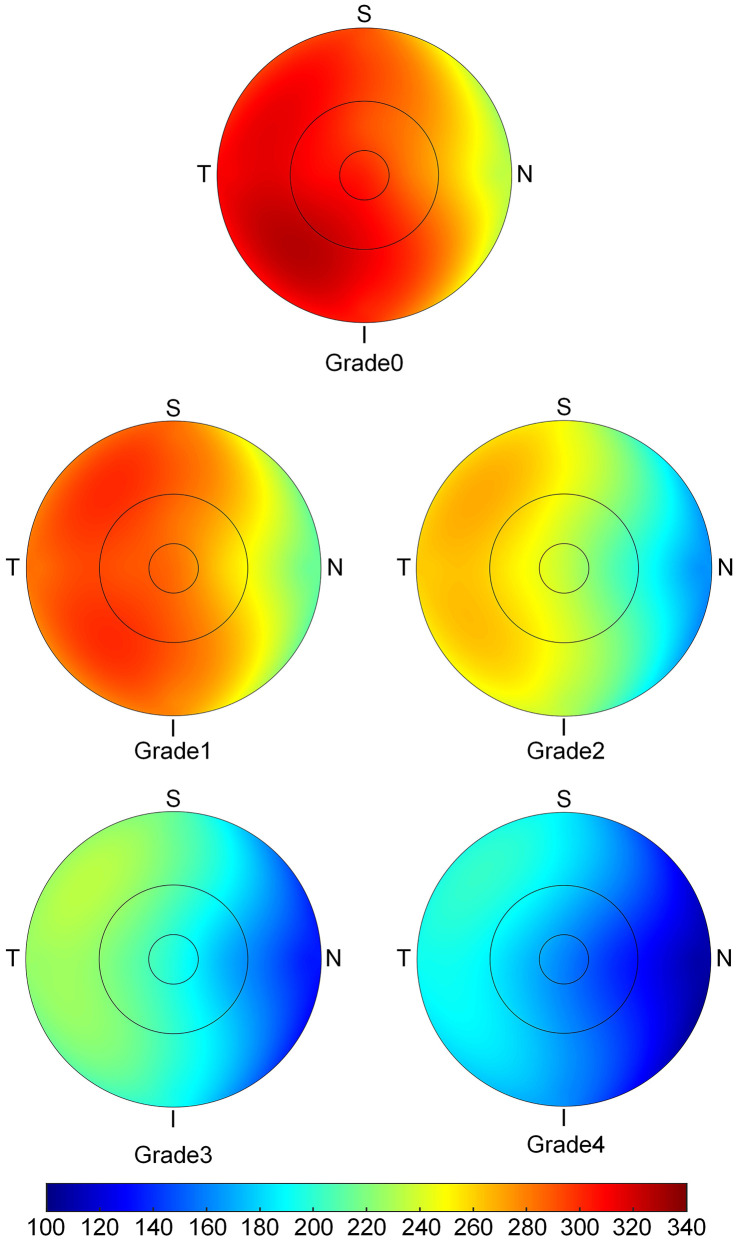
The topographic characteristics of choroidal layer thickness in ETDRS grid centered in fovea, from Grade 0 to Grade 4. The value of choroidal thickness was shown in different color, with color red being the thickest to color blue the thinnest.

**Figure 4:**
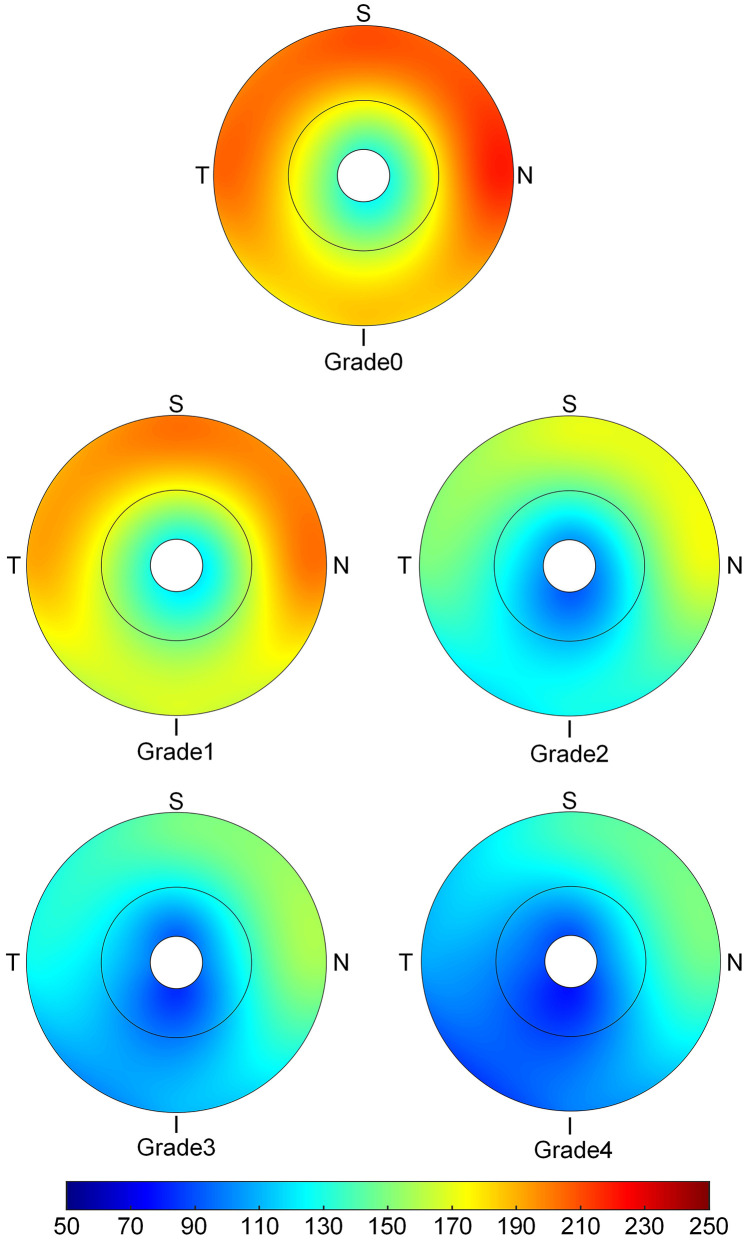
The topographic characteristics of choroidal layer thickness in ETDRS grid centered in optic disc range from Grade 0 to Grade 4. The value of choroidal thickness was shown in different color, with color red as the thickest to color blue the thinnest.

The topographic characteristics of choroidal layer thickness in macula and peripapillary tessellation ranging from Grade 0 to Grade 4 are shown in Table 4. There was a significant decrease in ChT of all 9 subregion of EDTRS grid (subfoveal, inner nasal, inner superior, inner temporal, inner inferior, outer nasal, outer superior, outer temporal, outer inferior) as the proportion of fundus tessellation enlarged toward center fovea. The trend of decrease of ChT in each subregion were shown in the linear graph (Figure 2). The data of ChT in each subregion of each grade were then converted to a choroidal thickness map (Figures 3, 4), where the value of ChT was shown in different color, with color red being the thickest to color blue the thinnest. As shown in the results of choroidal thickness map, center fovea, macular-papillary region and inferior region showed the most significant decrease in ChT as fundus tessellation enlarged.

**Table 4:** Topographical characteristics of choroidal and scleral thickness in tessellated fundus range from Grade 0 to Grade 4.

**Variable**	**Grade 0 (*N =* 78)**	**Grade 1 (*N =* 61)**	**Grade 2 (*N =* 220)**	**Grade 3 (*N =* 267)**	**Grade 4 (*N =* 170)**	**Statistic value**	***p*–value**
Average ChT, μm	296.02 ± 54.37	275.97 ± 57.35	234.02 ± 39.10	197.20 ± 35.06	165.21 ± 36.96	138.46	< 0.0001
Central fovea, μm	311.38 ± 63.53	292.21 ± 73.61	241.44 ± 48.72	201.12 ± 42.95	162.84 ± 45.29	120.7	< 0.0001
Parafovea temporal, μm	317.89 ± 68.98	298.17 ± 63.65	258.36 ± 50.76	219.76 ± 45.01	181.77 ± 45.79	98.34	< 0.0001
Perifovea temporal, μm	320.25 ± 68.85	291.22 ± 54.91	268.05 ± 50.38	230.89 ± 45.43	196.63 ± 45.08	79.02	< 0.0001
Parafovea Superior, μm	299.57 ± 70.52	291.04 ± 68.03	246.57 ± 52.07	209.65 ± 44.35	176.28 ± 43.70	96.34	< 0.0001
Perifovea Superior, μm	299.24 ± 65.45	292.31 ± 60.94	253.72 ± 50.45	218.40 ± 45.86	189.47 ± 44.42	86.25	< 0.0001
Parafovea Nasal, μm	287.24 ± 64.05	267.17 ± 69.12	213.80 ± 42.61	175.60 ± 38.94	141.53 ± 40.01	194.06	< 0.0001
Perifovea Nasal, μm	240.38 ± 54.60	219.30 ± 57.17	167.01 ± 36.42	135.82 ± 33.73	109.37 ± 34.43	211.62	< 0.0001
Parafovea Inferior, μm	321.03 ± 63.05	295.29 ± 77.66	246.62 ± 46.00	203.64 ± 40.08	164.21 ± 44.27	196.01	< 0.0001
Perifovea Inferior, μm	184.56 ± 47.75	166.68 ± 43.41	126.95 ± 32.41	110.63 ± 30.66	98.92 ± 30.37	202.88	< 0.0001
SST, μm	518.04 ± 37.33	515.38 ± 34.21	478.39 ± 35.17	471.29 ± 39.46	441.70 ± 54.52	24.73	< 0.0001

*ChT, choroidal thickness; SST, subfoveal sclera thickness*.

Logistic regression analyses were used to determine the factors that were strongly associated with the increase of proportion of fundus tessellation. First, we included all the participants without considering the data of scleral thickness, the results are showed in Table 5. The proportion of fundus tessellation significantly increased with lower BMI(*p* = 0.0067), longer AL (*p* < 0.0001), larger PPA (*p* = 0.0058), and thinner choroid (*p* < 0.0001) (Table 4). The data of each parameter were further divided and analyzed using interquartile range. Our results showed that for mChT, quartile 2 (*p* < 0.001; coefficient,–1.25; 95% Cl, 0.19–0.43), quartile 3 (*p* < 0.001; coefficient, −2.36; 95% Cl, 0.06–0.15), and quartile 4 (*p* < 0.001; coefficient, −3.49; 95% Cl, 0.02–0.05) all have lower risk of enlargement of fundus tessellation toward center fovea compared with quartile 1. For AL, quartile 3 (*p* = 0.0098; Coefficient, 0.51; 95% Cl, 1.13–2.44) and quartile 4 (*p* = 0.0003; Coefficient, 0.76; 95% Cl, 1.41–3.24) have higher risk of enlargement of fundus tessellation compared with quartile 1. For PPA, quartile 3 (*p* < 0.001; coefficient, 1.03; 95% Cl, 1.88–4.15) and quartile 4 (*p* < 0.001; coefficient, 0.84; 95% Cl, 1.52–3.51) have higher risk of enlargement of fundus tessellation compared with quartile 1. While for pChT, only quartile 4 (*p* < 0.001; coefficient, −1.20; 95% Cl, 0.19–0.48) showed a lower risk of enlargement of fundus tessellation compared with quartile 1.

**Table 5:** Associations of fundus tessellation severity with ocular and systemic parameters.

**Parameters**	**Regression coefficient**		***P*-value**	**OR (95% Cl)**
Gender		−0.1629	0.2814	0.85 (0.63, 1.14)
BMI		−0.0684	0.0067	0.93 (0.89, 0.98)
	Normal weight to underweight	−0.0688	0.6910	0.93 (0.67, 1.31)
	Overweight to underweight	−0.3552	0.1188	0.70 (0.45, 1.10)
AL		0.3002	< 0.0001	1.35 (1.17, 1.55)
	Quartile 2 to Quartile 1	0.2874	0.1355	1.33 (0.91, 1.94)
	Quartile 3 to Quartile 1	0.5074	0.0098	1.66 (1.13, 2.44)
	Quartile 4 to Quartile 1	0.7603	0.0003	2.14 (1.41, 3.24)
mChT		−0.0268	< 0.0001	0.97 (0.97, 0.98)
	Quartile 2 to Quartile 1	−1.2524	< 0.0001	0.29 (0.19, 0.43)
	Quartile 3 to Quartile 1	−2.3576	< 0.0001	0.09 (0.06, 0.15)
	Quartile 4 to Quartile 1	−3.4937	< 0.0001	0.03 (0.02, 0.05)
mReT		−0.00957	0.1222	0.99 (0.98, 1.00)
	Quartile 2 to Quartile 1	0.1839	0.3512	1.20 (0.82, 1.77)
	Quartile 3 to Quartile 1	−0.2876	0.1440	0.75 (0.51, 1.10)
	Quartile 4 to Quartile 1	−0.2556	0.2117	0.77 (0.52, 1.16)
pChT		−0.00537	0.0228	0.99 (0.99, 1.00)
	Quartile 2 to Quartile 1	−0.3092	0.1352	0.73 (0.49, 1.10)
	Quartile 3 to Quartile 1	−0.1566	0.4770	0.86 (0.56, 1.32)
	Quartile 4 to Quartile 1	−1.1988	< 0.0001	0.30 (0.19, 0.48)
PPA		1.9647	0.0058	7.13 (1.76, 28.84)
	Quartile 2 to Quartile 1	0.2273	0.2393	1.26 (0.86, 1.83)
	Quartile 3 to Quartile 1	1.0275	< 0.0001	2.79 (1.88, 4.15)
	Quartile 4 to Quartile 1	0.8377	< 0.0001	2.31 (1.52, 3.51)

*BMI, body weight index; AL, axial length; mChT, macular choroidal thickness; mReT, macular retinal thickness; pChT, peripapillary choroidal thickness; PPA, peripapillary atrophy*.

Second, we analyzed the data of eyes where scleral thickness was measurable. Among the 796 participants, 470 scleral thickness were measurable (59.05%) with the posterior scleral border adequately visible. Table 6 showed that for these participants, the proportion of fundus tessellation significantly increased with longer AL (*p* = 0.0159), thinner choroid (*p* < 0.0001) and thinner sclera (*p* = 0.0003).

**Table 6:** Associations of fundus tessellation severity with ocular and systemic parameters with scleral thickness.

**Parameters**	**Regression coefficient**	***P*-value**	**OR (95% Cl)**
Gender	0.0791	0.6931	1.08 (0.73, 1.60)
BMI	−0.0477	0.1420	0.95 (0.89, 1.02)
AL	0.3003	0.0015	1.35 (1.12, 1.62)
PPA	1.2140	0.1817	3.37 (0.57, 20.00)
mChT	−0.0306	< 0.0001	0.97 (0.96, 0.98)
mReT	−0.00945	0.2615	0.99 (0.97, 1.01)
pChT	−0.00107	0.7393	1.00 (0.99, 1.01)

*BMI, body weight index; AL, axial length; mChT, macular choroidal thickness; mReT, macular retinal thickness; pChT, peripapillary choroidal thickness; PPA, peripapillary atrophy*.

## Discussion

This is the first study of a university student population-based cohort to explore factors associated with tessellated fundus and its relative location with the fovea. In this study, we used a novel grading method to assess the severity of fundus tessellation, where the relative location between center fovea and the occurrence of fundus tessellation were taken into account. Compared with former studies which graded fundus tessellation severity by the visibility of large choroidal vessels in fundus photographs, this novel grading method has a clear standard by using ETDRS grid to determine the scope of fundus tessellation, thus can be more objective and practical in clinical practice (11, 12). The results of this study showed that the thicknesses of choroidal and scleral layers were significantly decreased, AL and PPA increased during the occurrence and enlargement of fundus tessellation toward center fovea. The thickness of retinal layer was also decreased in tessellated fundus compared to normal fundus, however, in the logistic regression analysis, the thinning of ReT was not a risk factor for the progression of fundus tessellation. In previous studies, the relationship between ReT and myopia remains controversial. Jin et al. studied the ReT in children with different refractive status and their results showed that myopic Chinese children have a thinner retina in the superior and inferior perifoveal regions than do their emmetropic and hyperopic counterparts (24). Jonas et al. studied the relationship of ReT and AL in 1,117 individuals with a mean age of 64.2 ± 9.7 years, their results showed that myopic axial globe elongation was associated with retinal thinning in the equatorial and pre-equatorial region, while foveal ReT was mostly unaffected by AL (25). Zhou et al. found that RNFL thicknesses were significantly thinner in high myopia compared to low myopia, except for the temporal quadrant (26). However, there is other study found no statistical different in the ReT between high myopia and normal population (27). Further investigation in ReT in subdivision of its location and layer are needed to reveal its relationship with fundus tessellation or other myopic retinopathy.

The results also showed a pattern of distribution in the decrease of ChT with higher degree of fundus tessellation, where the central fovea and the area between central fovea and optic disc showed the most significant decrease in ChT. The AL, choroidal and scleral thicknesses were independently related to macular fundus tessellation and its severity, while area of PPA and TD in addition to AL, choroidal and scleral thicknesses were independently associated with peripapillary fundus tessellation and its severity. Our results also showed that BMI could be a protective factor for the enlargement of fundus tessellation. We speculate that it can be associated with the tissular support effect and its function in preventing the deformation of the globe, yet further investigation with MRI is needed.

The results of the present study were in agreement with previous studies that reported a decrease in ChT with the progression of fundus tessellation and pathological myopia. Yan et al. (12) studied 3,468 participants with an average age of 64.4 years, and found that subfoveal ChT and AL had the strongest association with a higher degree of fundus tessellation. As compared to their data, our participants had similar ChT in grade 0 and grade 1 groups. However, the mean central foveal ChT of grade 2 and grade 3 in this study were 165 μm and 152 μm 152 μm as compared to 122 μm in grade 2 and 81 μm in grade 3 in the elderly people. might be due to age-related differences, as ChT is strongly age-related (14–17). Moreover, we noticed a pattern of ChT distribution where ChT in temporal areas was significantly thicker than that of the nasal areas, which is consistent with prior studies (12, 27, 28). The ChT in center fovea and the region between center fovea and optic disc showed the most significant decrease in ChT among all subfields of macular and peripapillary regions, which was consistent with a previous study on distribution pattern of ChT in Chinese children with myopia (29). We observed that the decrease in ChT became more significant with the progression of fundus tessellation.

Due to the limitations of technology and devices, the relationship between scleral thickness and myopia has not been extensively studied. In previous studies, Deng et al. (30) indicated that hyperopic and emmetropic children had thicker sclera than myopic children. In adults, previously reported rates of detection for the posterior scleral border with SS-OCT ranged from 53 to 84.7%, and the posterior scleral border was better seen as the AL increased (31–33). Our results showed a relevance ratio of 59.05% among university students, and indicated that eyes with fundus tessellation have a thinner SST than eyes with normal fundal appearance. Our results also showed that SST decreased significantly when fundus tessellation approaches to center fovea.

Parapapillary gamma zone atrophy is characterized by the absence of Bruch's membrane, retinal pigment epithelium, retinal deep layers and loss of choroicapillars (20–22). PPA is well-established in primary open-angle glaucoma (POAG) and its significance in progression of glaucomatous damage and visual field loss have been confirmed. Its association with myopia is a research hotspot. Several studies found that the area of PPA was strongly associated with longer AL, myopic refraction and tilted optic disc. An increase in AL and decrease in ChT might lead to a decrease in choroidal perfusion and eventually lead to atrophy of choroicapillars (34–36). Our study found that area of parapapillary gamma zone atrophy was strongly associated with fundus tessellation and its enlargement of proportion toward center fovea.

This study investigated optical parameters and changes in thickness of retina, choroid and sclera in the occurrence and increase of fundus tessellation proportion in young adults. The distribution of the decrease in ChT may refer to the deformation of globe and staphyloma formation, which can be further studied by MRI. This study also achieved a relatively high relevance ratio of scleral thickness measurement and observed a strong association between thinner sclera and higher degree of fundus tessellation, which offers certain clues about the mechanism of formation during myopic retinopathy. In this study, we can also found fundus tessellation in non-myopic (A L≤ 24) eyes, of which 75.47% are peripheral fundus tessellation (Grade0-2), while in high myopia (AL > 26), the ratio of fundus tessellation near center fovea (grade3-4) was 73.43%. This information indicate that fundus tessellation that involve center fovea could be more significative in further study of myopic retinopathy.

This study had several limitations. First, since this study exclusively included university students as participants, the results may not represent the general population of all young adults. Second, the novel grading method for tessellated fundus still has subjective element as the relative location of fundus tessellation was determined by the examiner using ETDRS grid instead of a total objective quantitative method. Third, the SST was only measured when the posterior scleral border was clear, which means a greater loss of data in grade 0 and grade 1 tessellated fundus due to thicker choroidal and scleral layers. Due to incomplete data in eyes with unclear scleral borders, we may have underestimated the value of scleral thickness in emmetropia and low myopia participants, however, our results reveal an important tendency that SST decreased significantly with the progression of tessellated fundus.

In summary, this study suggested that BMI, AL, PPA, choroidal, and scleral thicknesses were closely correlated with fundus tessellation in Chinese young adults. Choroidal thinning may progress faster in the region between central fovea and optic disc as the fundus tessellation evolves to more severe complications of pathological myopia. The PPA and TD may play an important role in the evolvement of pathological myopia. The relative location between fundus tessellation and center fovea could be an ideal standard to assess the severity of fundus tessellation and those who has fundus tessellation involving center fovea (Grade 4) should be closely supervised as they showed the most severe myopic morphological alternations and may have more risks to progress into more severe myopic maculopathy. Tessellated fundus is the first stage of fundus changes during myopic maculopathy, whereby some patients will progress from diffuse atrophy to macular atrophy that causes severe and irreversible impairment to visual acuity, while others could remain stable at this stage forever. Thus, we will continue with follow-up visits of our participants, and further investigation of the progression of tessellated fundus and more severe impairment.

## Data Availability Statement

The raw data supporting the conclusions of this article will be made available by the authors, without undue reservation.

## Ethics Statement

This cross-sectional population-based study was approved by the ethics committee of Shanghai General Hospital, Shanghai Jiao Tong University, Shanghai, China, and followed the tenets of the Declaration of Helsinki. All participants understood the study protocol and provided signed informed consents.

## Author Contributions

HL have made substantial contributions to the conception or design of the work and the acquisition, analysis, or interpretation of data for the work. QC have made contributions to the acquisition of data and the supervision of the analysis of the data. GH have made contributions to the acquisition of data and data processing work. YS and LY have made contributions to the revise work of this article. YY have made contributions to the acquisition of data and technical support. YF have made contributions to the supervision of the analysis of the data. HZ have made contributions to the supervision of the analysis of data and the writing. JH, JZ, and XX have made contributions to the supervision of the article writing and approved the final version to be published. All authors contributed to the article and approved the submitted version.

## Conflict of Interest

The authors declare that the research was conducted in the absence of any commercial or financial relationships that could be construed as a potential conflict of interest.

## Abbreviations

ACD: anterior chamber depth
AL: axial length
BCVA: best-corrected visual acuity
ChT: macular choroidal thickness
CI: confidence interval
TD: tilted disc
mChT: macular choroidal thickness
GCL: ganglion cell layer
mReT: macular retinal thickness
mRNFLT: macular retinal nerve fiber layer thickness
OR: odds ratio
pChT: peripapillary choroidal thickness
pGCLT: peripapillary ganglion cell layer thickness
PPA: parapapillary atrophy
pReT: peripapillary retinal thickness
pRNFLT: peripapillary retinal nerve fiber layer thickness
SE: spherical equivalent
Ref: reference
SBP: systolic blood pressure
SST: subfoveal scleral thickness.
